# The rapid scale up of medical education in Ethiopia: Medical student experiences and the role of e-learning at Addis Ababa University

**DOI:** 10.1371/journal.pone.0221989

**Published:** 2019-09-05

**Authors:** Caitrin M. Kelly, Holly Vins, Jennifer O. Spicer, Brittney S. Mengistu, Daphne R. Wilson, Miliard Derbew, Abebe Bekele, Damen Haile Mariam, Carlos del Rio, Russell R. Kempker, Dawn L. Comeau, Henry M. Blumberg

**Affiliations:** 1 Department of Medicine, Emory University School of Medicine, Atlanta, GA, United States of America; 2 Department of Behavioral Sciences and Health Education, Rollins School of Public Health of Emory University, Atlanta, GA, United States of America; 3 Division of Infectious Diseases, Department of Medicine, Emory University School of Medicine, Atlanta, GA, United States of America; 4 School of Law, Emory University, Atlanta, GA, United States of America; 5 Department of Surgery, Addis Ababa University School of Medicine, Addis Ababa, Ethiopia; 6 Department of Community Health, School of Public Health, College of Health Sciences, Addis Ababa University, Addis Ababa, Ethiopia; 7 Hubert Department of Global Health, Rollins School of Public Health of Emory University, Atlanta, GA, United States of America; 8 Department of Epidemiology, Rollins School of Public Health of Emory University, Atlanta, GA, United States of America; University Lyon 1 Faculty of Dental Medicine, FRANCE

## Abstract

**Background:**

In response to a physician shortage in Ethiopia, the number of medical students admitted to public universities was rapidly increased through a “flooding” policy.

**Objectives:**

To assess medical student perceptions on the impact of the “flooding” policy on medical education and e-learning initiatives, as well as plans for future emigration.

**Design:**

A cross-sectional survey of medical students at AAU was implemented in 2014. Attitude and practice items were assessed using a Likert scale. Logistic regression analysis was performed to identify characteristics associated with an interest in future emigration.

**Results:**

673 (99.6%) of 676 students approached completed the survey, representing 39.5% of all 1705 medical students enrolled at AAU in 2014. Most students felt the “flooding” policy had a negative impact on their medical education and >90% felt there was not adequate infrastructure to support the increased student body. E-learning activities to accommodate increased class size included distribution of electronic tablets, but at the time of the survey only 34.8% of students still had a working tablet and 82.3% reported problems with internet connectivity. Most preclinical students (85.1%) who had attended live-streamed lectures preferred traditional classroom lectures. Half of the students (49.5%) intended to practice medicine in Ethiopia. Independent risk factors for planning to emigrate included age <21 years (aOR = 1.30, 95% CI 1.04, 1.97); having applied to medical school for reasons other than “wanting to be a physician” (aOR = 1.55, 95% CI 1.14, 2.20), and not believing that “flooding” policy would increase the number of physicians working in Ethiopia (aOR = 1.87, 95% CI 1.33, 2.58).

**Conclusions:**

The “flooding” policy lead to significant educational challenges that were not fully alleviated by e-learning initiatives. Concomitant increases in resources for infrastructure development and faculty expansion are needed to maintain quality medical education. Additional research is needed on factors that influence medical graduates decision to emigrate.

## Introduction

Ethiopia has had one of the most severe physician shortages in Sub-Saharan Africa with only 2.5 physicians per 100,000 people in 2009 [1]. The World Health Organization (WHO) recommends a minimum of ten physicians per 100,000 in low-income countries [2]. This physician shortage in Ethiopia is driven by multiple factors, including a limited number of medical schools and emigration of Ethiopian medical school graduates to countries with higher wages, a trend commonly referred to as “brain-drain” [3]. In order to address the physician shortage, the Ethiopian Federal Ministry of Health (FMOH) and Federal Ministry of Education (FMOE) initiated a “flooding” policy or “rapid scale-up,” that required existing public medical schools in Ethiopia to expand their enrollment three to fourfold, and opened 18 new medical schools between 2005 and 2009 [4,5]. Since the “flooding” policy was initiated, overall enrollment at Addis Ababa University (AAU) School of Medicine (SOM) increased markedly from 250 to over 1,700 students, or from less than 100 to over 300 medical students per class [4]. The rapid increase in medical student enrollment presented many challenges because infrastructure, faculty, and educational resources were not increased to match the expanded class size [4]. For example, after the expansion there were not enough seats in the largest lecture hall for all of the students in each of the preclinical classes, and the textbook-to-student ratio was approximately 1:15 [4].

E-learning, defined as any teaching or learning activity that incorporates Information Communication Technology (ICT), has been advocated by the WHO as a method to support training healthcare workers in the setting of faculty shortages in low and middle-income countries [6]. As part of efforts to respond to the increase in the number of medical students at AAU, e-learning strategies were introduced with funding from the U.S.-government supported Medical Education Partnership Initiative (MEPI) grant [7]. E-learning initiatives included installing and upgrading wireless internet connections, creating a computer lab with desktop computers, and purchasing equipment to live-stream lectures in an overflow lecture hall for students who did not have a seat in the main lecture hall. In addition, medical students were given an Android touchscreen UbiSlate tablet loaded with digital textbooks and eGranary software to improve access to learning materials and in the setting of a textbook shortage. EGranary is a digital library with free access to websites, books, academic journals, and multimedia resources via a local area network [8].

The AAU School of Medicine is a 6-year undergraduate medical school consisting of two preclinical years and four clinical years. Students matriculate following completion of high school. The third clinical year is called the “qualifier” year because students sit for national qualifying exams, after which they start their 6^th^ and final year, referred to as internship. After completing internship year and graduating, all students at public medical schools are required to complete a 2–4 year service period as a general practitioner in Ethiopia.

The purpose of our study was to assess medical student perceptions on both the impact of the “flooding” policy on medical education and on e-learning initiatives in response to larger classes, as well as to explore the potential impact on the physician shortage through student plans for future emigration. The study was initated in response to AAU faculty concerns about how the flooding policy might effect the quality of medical school education. This investigation is important for medical school faculty and policymakers considering approaches similar to the “flooding policy” to alleviate physician shortages.

## Methods

We performed a cross-sectional survey of medical students at AAU in Addis Ababa, Ethiopia, from June to July of 2014. Survey content was created from informal discussions and qualitative interviews with faculty and students, which was conducted as part of the same investigation [9]. The survey was piloted by 11 preclinical and clinical medical students to identify problems with survey instructions and content. Minor revisions were made based on comments written on the survey, as well as individual debriefing with students after piloting. The survey was conducted in English, the language of instruction at the AAU School of Medicine. The final survey consisted of 77 items for pre-clinical medical students (years 1 and 2), with an additional 26 items for medical students in the clinical years (years 3–6). The survey (Appendix 1) included sections on demographics, the “flooding” policy, preclinical classroom experiences, and e-learning initiatives. Items were assessed using multiple choice or a five-point Likert scale (strongly disagree, disagree, neither agree nor disagree, agree, strongly agree). The survey also included one open-ended question to collect qualitative data on student’s perceptions of how medical education at AAU could be improved. The survey was administered to AAU students in all six years of medical school. Convenience sampling was used to recruit students from lectures, seminars, meetings, and the clinical wards at Black Lion Hospital, the primary AAU teaching hospital. All participants gave oral consent to participate and all survey responses were anonymous. Data were entered into a REDCap database [10]. Data analyses were performed using SAS software (version 9.3) [SAS Inc., Cary, NC USA]. The study protocol was reviewed by the Emory University and AAU Investigational Review Boards (IRBs) prior to study initiation and determined to be exempt.

For analysis, all Likert scale responses were collapsed into dichotomous categories (agree/strongly agree vs. neither agree nor disagree, disagree, strongly disagree) in order to make the results more easily interpreted. Missing data for individual survey items were excluded from analysis. Outcomes included descriptive statistics of demographics and survey responses. McNemar’s test of proportions was used to compare student experiences in the traditional lecture classroom to experiences in the live-streamed classroom, as measured by Likert scale responses. Univariate analysis was conducted with a student’s interest in pursuing a career outside of Ethiopia as a binary outcome. Multivariate logistic regression models were constructed to assess characteristics associated with intending to work outside of Ethiopia using the purposeful selection of covariates strategy [11] and selection of variables based on biologic plausibility. A p-value ≤ 0.05 was considered statistically significant. In addition to the quantitative analysis, two members of the research team independently reviewed the responses to the open-ended question, “how can your medical education be improved?” and developed deductive codes with definitions to identify the emerging themes in the data. Themes directly related to education and technology are included in the results to provide context to the quantitative data; the remainder of the qualitative data has been previously reported [9].

## Results

### Demographics

Six-hundred-seventy-six medical students were recruited to participate in this study and asked to complete the anonymous survey. All students agreed to participate, but 3 students did not fill out the survey and were therefore excluded. A total of 673 (39.5%) of 1705 medical students at AAU completed the survey (Table 1). The mean age of medical students completing the survey was 21.1 years, and 457 (67.9%) were male. This gender distribution among those who completed the survey is reflective of the overall gender distribution at AAU, where approximately 70% of medical students are male. Additional characteristics of the study population are summarized in Table 1.

**Table 1:** Characteristics of medical student survey participants at the Addis Ababa University School of Medicine.

Characteristic	Value, no. (%) Total (n = 673)
Age, mean (SD), years	21.1 (2.0)
Female	216 (32)
Male	457 (67.9)
Married	8 (1.2)
**Year in medical school**	
Preclinical 1 (Year 1)	158 (23.5)
Preclinical 2 (Year 2)	146 (21.7)
Clinical 1 (Year 3)	114 (16.9)
Clinical 2 (Year 4)	57 (8.5)
Clinical 3 (Year 5)	118 (17.5)
Internship (Year 6)	80 (11.9)
Region of Ethiopia where the participant completed high school*	
Addis Ababa	382 (56.8)
Amhara Region	87 (12.9)
Oromia Region	87 (12.9)
SNNPR**	73 (10.9)
Other***	44 (6.5)
Reason for attending medical school*	
“I want to be a doctor”	397 (60.5)
Other Reasons:	259 (39.5)
“I had high exam scores”	76 (11.6)
“My parents want me to be a doctor”	50 (7.6)
“I want a high paying job”	46 (7.0)
“Could not pursue my first career choice”	23 (3.5)
Other	64 (9.8)

*Missing data = 17

**SNNPR—Southern Nations, Nationalities, and Peoples' Region

***Other includes students who selected multiple answers

### The “flooding” policy

The majority of medical students (427/666 or 64.1%) believed the “flooding” policy implemented by the FMOH will increase the number of physicians working in Ethiopia (Table 2). However, only a minority of medical students (154/668, 23.1%) agreed that the rapid scale up of medical students AAU was good policy, and 92.1% believed that it was difficult for AAU to support the increased number of students. More than two-thirds of medical students believed that was difficult to learn due to the large number of students in class following implementation of the “flooding” policy (Table 2).

**Table 2:** Student attitudes toward the “flooding” policy.

Statement	Likert Scale Responses in Agreement, No. (%) Total (n = 673)
There is a shortage of physicians in Ethiopia (missing = 6)	617 (92.5)
The government policy of increasing the number of students admitted to medical school will increase the number of physicians working in Ethiopia (missing = 10)	427 (64.1)
Increasing the number of students at AAU School of Medicine is a good policy (missing = 8)	154 (23.1)
It is difficult for AAU to support the increased number of medical students (missing = 7)	616 (92.1)
It is difficult for me to learn due to the large number of students in my class (missing = 12)	452 (68.1)
I would study more if I didn't have to share textbooks (missing = 10)	295 (44.3)
AAU does not have enough textbooks for medical students (missing = 10)	430 (64.6)

### E-learning and technology initiatives

A total of 656 (97.5%) students surveyed reported being given a tablet (Table 3). Among the 656 students who received a tablet, 228 (34.8%) reported that they still have the tablet and it works; 270 (41.2%) reported that the tablet had broken, stopped working, or functioned poorly, and 142 (21.6%) sold the tablet or gave it away. Reasons why students did not use the tablet (multiple responses permitted) are summarized in Table 3. Students who attended a workshop on use of the electronic tablet were more likely to have functioning tablets compared to those who did not attend a workshop (86/207, 41.5% vs 84/291, 28.9%, p<0.01). Most medical students (540/667, 81.0%) reported owning a personal laptop computer and 421/662 (63.6%) preferred to use a laptop for school. When asked to compare hard copy (paper) textbooks to electronic textbooks, 279/667 (41.8%) students noted a preference to use a hard copy textbook to study (Table 3). In the open response question, requests for a higher quality tablet were common, as were suggestions to provide laptops instead of tablets, as well as textbooks. One student in the open-ended question noted that they were appreciative of the electronic tablets for medical students but indicated that, “In addition to the tablet, we also need hard cop[ies]…of textbooks because [we didn’t learn] through tablets at high schools. We are adapted to books.”

**Table 3:** E-learning initiatives: Medical student tablet use and learning preferences.

Tablet use and learning preferences indicated by multiple choice responses	No. (%) Total (n = 673)
Received an electronic tablet (missing = 4)	656 (97.5)
Attended a tablet training session (missing = 13)	304 (46.1)
Own a personal laptop computer (missing = 6)	540 (81.0)
**Prefer to use a laptop or tablet for school** (missing = 14)	
I prefer to use a laptop	421 (63.6)
I prefer to use a tablet	172 (26.0)
No preference	69 (10.4)
**Prefer to use a textbook or electronic text** (missing = 9)	
I prefer to use a hard copy textbook	279 (41.8)
I prefer to use an electronic text	291 (43.6)
I do not have a preference	97 (14.5)
Primary use of tablet* (missing = 49)	
Medical school reading	475 (78.2)
Entertainment	47 (7.8)
Social Media	10 (1.7)
Other	75 (12.4)
Status of electronic tablet* (missing = 0)	
I still have tablet and it works	228 (34.8)
Tablet broke, stopped working, or functions poorly	270 (41.2)
I sold the tablet or gave it away	142 (21.7)
The tablet was lost or stolen	16 (2.4)

*n = 656, the number of participants who received a tablet

Through the MEPI grant, wireless internet on the AAU College of Health Sciences campus which includes the School of Medicine was upgraded from 6 megabits-per-second (mbps) to 50 mbps and expanded to include student dormitories and Black Lion Hospital. Despite the upgrade, challenges connecting to the internet were reported in the survey (Table 4). The need for improved internet access emerged as a common theme in the open response question as well. One student commented, “The internet connection is so poor. The money being spent on it is wasted as we students are not able to use the internet due to technical problems.”

**Table 4:** E-learning initiatives: Student use of internet and campus computers.

In the past month….	Likert Scale Responses in Agreement, No. (%) Total (n = 673)
I needed to use the internet for class (missing = 43)	460 (72.7)
The internet on campus was too slow to be useful (missing = 49)	516 (82.3)
I could not connect to the internet on campus (missing = 48)	455 (72.5)
I was able to access PowerPoint presentations from lectures through the internet (missing = 83)	146 (24.8)
There was a computer on campus for me to use when needed (missing = 94)	171 (29.5)

Live-streamed lectures or “smart classes” were launched in January 2014 for preclinical classes in response to the insufficient number of seats in the main lecture hall following the “flooding” policy. The “smart” classroom functions as an overflow room, where lectures are projected on a screen, in real time in an adjacent lecture hall. Two-hundred-twenty-two (73.0%) of the 304 preclinical students who completed the survey reported having attended a “smart class.” The large majority of preclinical students (189/219 or 86.3%) preferred to attend traditional lectures rather than “smart” classes. Among 222 preclinical students who had experienced both “smart” classrooms and traditional classrooms, a significantly higher proportion agreed that they were able to hear (151/208, 73%), learn (156/207, 75%), and ask questions (140/206, 68%) in a traditional classroom compared to the proportion of students who were able to hear (83/207, 40%), learn (60/207, 29%) and ask questions (33/206, 16%) in the “smart” classroom (Table 5).

**Table 5:** Comparison of student attitudes toward the “smart*” classroom and traditional classroom measured by likert scale responses.

Statement	Proportion of Likert Scale Responses in Agreement, No. (%) (n = 222**)			
	Traditional Class	Smart* class	Odds Ratio	McNemar Two- tailed p-value
I am able to hear the instructor	151/208 (73)	83/208 (40)	4.4	<0.001
I am able to learn during class	156/207 (75)	60/207 (29)	5.0	<0.001
I am able to ask instructors questions	140/206 (68)	33/206 (16)	27.8	<0.001

* “Smart” classrooms refer to overflow classrooms where lectures were streamed live on a projector screen for students who did not have a seat in the traditional lecture classroom.

** n = 222, the number of preclinical students who reported having attended both lecture in both the “smart” classroom and traditional classroom; missing data accounts for difference between listed denominators.

The “smart classes” were also mentioned numerous times in the open-ended question with students commenting on the difficulty absorbing lecture material due to audio-visual malfunctions and other students talking. One student wrote, “the use of smart classes seems efficient but … equipment malfunctions and inaudible sound (from the lecturer) make it extremely difficult to learn in.” Another student commented, “Smart class is not actually a class, because if the traditional class doesn’t have a space and we go to the smart class, what we do is play a game or watch movie and that’s because we can’t hear what the instructor is saying.”

### Post-graduation career plans

49.5% of medical students (325/656) indicated that they were planning to practice medicine in Ethiopia after medical school while 164/656, (25.0%) were planning to practice medicine outside of Ethiopia and 167/656 (25.5%) were undecided. Univariate analysis of risk factors for planning to practice medicine outside of Ethiopia are shown in Table 6.

**Table 6:** Univariate analysis: Predictors of intention to pursue a medical career outside ethiopia among medical students at Addis Ababa University.

Characteristic	Total (n = 656)	Intends to Practice in Ethiopia (n = 325)	Intends to Practice Outside Ethiopia or Undecided (n = 331)	Odds Ratio, 95% CI.
**Age**				** **
21 years and older	283(43.1)	198 (60.9)	175 (52.9)	0.72 (0.53, 0.98)
Less than 21 years	373 (56.9)	127 (39.1)	156 (47.1)	
**Gender**				
Male	445 (67.8)	227 (69.9)	218 (65.9)	
Female	211 (32.2)	98 (30.2)	113 (34.1)	0.83 (0.60, 1.16)
**Location of Secondary School**				
Addis Ababa	374 (57.0)	171 (52.6)	203 (61.3)	
Outside of Addis Ababa	282 (43.0)	154 (47.4)	128 (38.7)	1.43 (1.05, 1.95)
**Primary reason for applying to medical school**				
I want to be a doctor	393 (60.4)	212 (65.6)	181 (55.2)	
Other*	258 (39.6)	111 (34.4)	147 (44.8)	0.64 (0.47, 0.88)
**Year category**				
Preclinical	296 (45.1)	140 (43.1)	156 (47.1)	
Clinical	360 (54.9)	185 (56.9)	175 (52.9)	1.18 (0.87, 1.60)
**Intended Area of Practice**				
Primary Care**	178 (39.9)	93 (40.8)	85 (39.0)	
Other***	268 (60.1)	135 (59.2)	133 (61.0)	0.93 (0.63, 1.36)
**Identified physician ratio in Ethiopia**				
Correctly	286 (44.9)	150 (47.8)	136 (42.1)	
Incorrectly/Do Not Know	351 (55.1)	164 (52.2)	187 (57.9)	0.80 (0.58, 1.09)
**Policy will increase doctors in Ethiopia**				
Agree	416 (63.9)	229 (71.1)	187 (56.8)	
Disagree/neutral	235 (36.1)	93 (28.9)	142 (43.2)	0.53 (0.39, 0.74)
**Research opportunities are available**				
Agree	106 (16.5)	60(18.7)	46 (14.2)	
Disagree/neutral	538 (83.5)	261 (81.3)	277 (85.8)	0.72 (0.47, 1.10)
**Interested in Research**				** **
Agree	421 (65.2)	206 (64.4)	215 (66.0)	
Disagree/neutral	225 (24.8)	114 (35.6)	111 (34.1)	1.07 (0.78, 1.48)
**Satisfied with preclinical education**				
Agree	116 (18.1)	60 (18.9)	56 (17.2)	
Disagree/neutral	526 (81.9)	257 (81.1)	269 (82.8)	0.89 (0.60, 1.33)
**Instructors value mentoring**				
Agree	153 (23.8)	83 (25.9)	70 (21.7)	
Disagree/neutral	490 (76.2)	237 (74.1)	253 (78.3)	0.79 (0.55, 1.14)
**Class size**				** **
large class (>300)	332 (74.4)	168 (75.7)	164 (73.2)	
smaller class size (182)	114 (25.6)	54 (24.3)	60 (26.8)	0.88 (0.57, 1.35)

* Other includes: My parents want me to be a doctor, I had high exam scores, I want to have a high paying job, I could not pursue my first career choice

**Primary care includes Internal Medicine, Family Medicine, General Practitioner, Pediatrics, OB/GYN

***Other includes: surgery, otolaryngology, ophthalmology, radiology, psychiatry, public health, dermatology, emergency medicine, undecided

In multivariate analysis, medical students < 21 years of age (aOR = 1.30, 95% CI 1.04, 1.97), those students who applied to medical school for reasons other than wanting to be a physician (aOR = 1.55, 95% CI 1.14, 2.20), and those who did not believe that the “flooding” policy would increase the number of physicians working in Ethiopia (aOR = 1.87, 95% CI 1.33, 2.58) were significantly more likely to indicate that they were either considering or planning to leave Ethiopia following medical school (Table 7).

**Table 7:** Multivariate analysis of predictors of intention to pursue a medical career outside of ethiopia among medical students at Addis Ababa University.

	Univariate Analysis		Multivariate Logistic Regression	
Variable	Odds Ratio	p-value	Odds Ratio	95% CI
**Age Category (years)**	** **	** **		** **
Age ≥21	1.00		1.00	
Age < 21	1.30	0.04	1.43	1.04–1.97
**Gender**				
Male	1.00		1.00	
Female	1.20	0.28	1.07	0.75–1.54
**Reason for applying to medical school**				
Want to be a doctor	1.00		1.00	
Other*	1.55	<0.01	1.59	1.14–2.20
**Where completed high school**				
Outside of Addis Ababa	1.00		1.00	
Addis Ababa	1.43	0.02	1.36	0.97–1.91
**“Flooding” policy will increase physicians working in Ethiopia**				
Agree	1.00		1.00	
Disagree/neutral	1.87	<0.01	1.85	1.33–2.58

*Other reasons include: My parents want me to be a doctor, I had high exam scores, I want to have a high paying job, I could not pursue my first career choice, other

## Discussion

In an effort to address one of the most severe physician shortages in the world and pursue the goal of universal primary health coverage, Ethiopia launched an unprecedented increase in medical school enrollment as part of the “flooding” strategy. Through a cross-sectional survey, we hoped to generate hypotheses on how the “flooding” policy might impact the quality of medical education and the physician shortage. Most students felt the rapid scale-up of medical students at AAU had a negative impact on their medical education and >90% felt that AAU did not have adequate infrastructure to support the increased number of medical students. Perhaps more moderate or gradual increases in class size would allow for concurrent expansion of resources and infrastructure.

Students also perceived several challenges with the e-learning initiatives. Only one-third of the electronic tablets distributed were still being used by the students, and most students were either not aware of or not using the eGranary digital library at the time of the survey. A vast majority of students preferred traditional lecture halls to overflow “smart” classrooms with live video streaming due to decreased ability to engage with professors and hear the lecture material. Despite MEPI-funded upgrades to the internet connectivity and bandwidth at the AAU College of Health Sciences, most medical students (>80%) reported that the internet connectivity was poor.

MEPI has helped to support medical education through collaborative relationships and e-learning initiatives throughout Sub-Saharan Africa [7,12]. The experience and perceptions of e-learning initiatives may vary between institutions in Africa, and the experience at AAU has likely been impacted by the rapidity and degree of the scale up of medical student class size. In comparison, a MEPI grant was used at Kilimanjaro Christian Medical University College (KCMUC) in Tanzania to install a fiber-optic cable to improve internet access, procure computers, and purchase tablets with curricular materials for all students. The funding was also used to install a learning management system (LCMS+) that had been previously used and optimized for over a decade at Duke University School of Medicine. An evaluation of these initiatives found that 90% of first year medical students at KCMUC accessed the online curriculum frequently one year after its implementation [12]. While there had been an increase in the number of medical student at KCMUC (from 15 to 155 per class) it took place over more than a decade, while at AAU the increase of more than 250 students per class (from <100 up to 350) took place within only a few years. These differences and ongoing challenges in wireless internet connectivity at AAU may have led to the lower levels of utilization reported by students in the survey.

Our survey suggests that the impact of the roll out of tablet-based e-learning at AAU was limited for several reasons. First, the tablet technology may not have been as urgently needed as initially anticipated. The finding that >80% of AAU medical students surveyed owned a personal laptop was unexpected, and indicates the importance of conducting pre-implementation baseline surveys. In addition, the unreliability of the tablet and poor wireless internet access on the medical school campus also may have limited the usefulness of the tablets. Piloting the use of the tablet, as well as the “smart” classroom could have identified issues in advance. Interestingly, students who attended training workshops on the use of the tablet were significantly more likely to have a functioning tablet than those who did not attend the training, emphasizing the importance of training students on new technologies being implemented. This association could also reflect a selection bias in that students who attended a non-mandatory training may have also had characteristics that led to more conscientious care of a tablet. E-learning at AAU has been impacted by challenges also recognized at institutions in other low and middle-income countries; these include electicity failures and bandwidth limitations causing slow internet with low quality and slow downloading [6]. Slow internet connection speeds have been shown to be a barrier to accessing information in Ethiopia and other East African countries [13]. However, internet based e-learning in Ethiopia may be more successful in the future. Bandwidth is expected to increase substantially in Ethiopia with the development and implementation of the Gulf to Africa (G2A) submarine cable [14].

Loss of intellectual capital or “brain drain” has been an important contributing factor to the shortage of physicians in Sub-Saharan Africa including Ethiopia [15–17]. Despite the “flooding” policy, between 2004–2009 a majority of the new surplus of medical graduates were lost to other countries or urban areas within Ethiopia [4]. When surveyed about their plans for where they plan to practice medicine following completion of medical training, about half of AAU medical students planned to practice in Ethiopia and the other half either planned to practice outside of Ethiopia or were undecided and considering that possibility. These career intentions are similar to a those reported in a cross-sectional study of 251 medical students in Uganda, where 44.6% of graduating students wanted to leave Uganda to work elsewhere [17]. We identified independent risk factors associated with planning to leave Ethiopia following medical school; these included age <21 years (OR = 1.30), applying to medical school for reasons other than “wanting to be a physician” (OR = 1.55), and not believing that the scale up of medical students would increase the number of physicians working in Ethiopia (OR = 1.87). A previous study in Ethiopia found a high level of “intrinsic motivation” among healthcare workers deciding to work in the public sector or in rural areas [18]. On our survey, students who reported “wanting to be a doctor” as the primary reason for applying to medical school may be relective of having higher “intrinsic motivation,” and may be more likely work in Ethiopia after graduation. A systematic review on factors influencing medical students’ motivation to practice in rural areas in low-income and middle-income countries identified being brought up in a rural area, training in rural areas and community exposure as significant motivating factors for students to work in rural areas [19]. Our study demonstrated that students from Addis Ababa, the largest urban area in Ethiopia, had higher odds of wanting to work outside of Ethiopia, however this was not statistically significant (C.I. 0.97–1.91). Further research is needed on motivating factors to stay and work in Ethiopia if the flooding policy is to be effective. These factors could generate new medical school admission selection metrics or processes, which are currently based exclusively on academic achievement as measured by national exam scores. This could have a major impact on physician shortages in Sub-Saharan Africa as more schools undergo expansion. The Sub-Saharan African Medical Schools Study (SAMSS) found that 76% of medical schools surveyed had increased enrollment in the past 5 years, and 53% had plans to increase enrollment over the next 5 years, many mandated by government policy [20]. It will be important to develop a system to track cohorts of medical graduates from AAU and other Ethiopian medical schools to assess the continued impact of the “flooding” policy on retention of physicians in Ethiopia.

## Limitations

Our study was subject to several limitations. For feasibility reasons, we used a convenience sampling strategy rather than surveying a random sample of AAU medical students. Convenience sampling resulted in lower levels of participation from students in clinical year 2 and internship due to these students participating in clinical rotations at multiple hospitals across Addis Ababa and not being available for recruitment at the primary teaching hospital, Black Lion Hospital. However, essentially all students approached agreed to participate in the study, and our survey included participants from each class in the 6-year medical school at AAU. Our study was also limited by not having previously validated scales to assess metrics of interest. Because of the uniqueness of the situation we were assessing, there were no previously developed standardized surveys. This led us to develop our own survey instrument. Further study instruments need to be designed and validated for evaluating other medical schools undergoing rapid expansion. Despite these limitations, we believe we were able to design an instrument that was useful for a preliminary exploratory investigation at AAU.

## Conclusions

The shortage of physicians in Sub-Saharan Africa and other areas is a major global public health problem that impacts the ability to deliver effective healthcare. Ethiopia, with one of the lowest physician to population ratios, has undergone an unprecedented scale of up of medical students in an effort to address the severe physician shortage and provide universal health care. This has greatly impacted medical education at AAU and other medical schools in Ethiopia. Expanding enrollment in medical schools is a strategy being used by many countries to address physician shortages. Our study findings provide important lessons learned for other medical schools and governments in low-income countries also undergoing enrollment expansion and implementing e-learning initiatives. Before e-learning initiatives are implemented, we recommend first conducting a baseline survey to assess the prevalence of student tablet and laptop ownership, student preferences for technology and learning resources, internet access, availability of outlets for charging, and faculty attitudes toward e-learning. An extended pilot period for e-learning initiatives is also recommended to ensure quality and functionality before new initiatives are fully rolled out. We believe lack of internet connectivity had a major impact upon the e-learning programs at AAU, however initiatves such as the G2A may enhance future technology innovation by providing enhanced internet connectivity to East Africa. In addition, training workshops for students, faculty and information technology staff are likely to be an important program component to ensure e-learning success. Furthermore, brain-drain has greatly contributed to physician shortages in Ethiopia and throughout Sub-Saharan Africa. We identified several independent risk factors associated with medical students planning to leave Ethiopia following medical training, however more research is needed in this area. Despite some initial challenges in the implementation of e-learning activities, we recommend continued strengthening of medical education in Ethiopia and Sub-Saharan Africa and further exploration of technology to improve education. Our findings support the importance of increased investment in faculty and medical school infrastructure to promote quality medical education and retention of the next generation of Ethiopian physicians.

## Supporting information

S1 Dataset Excel spreadsheet of survey data. (XLS)
